# Determinants of counseling on neonatal danger signs among women in Somalia: a multilevel mixed-effects logistic regression analysis of the 2020 SDHS

**DOI:** 10.3389/fped.2026.1844879

**Published:** 2026-05-29

**Authors:** Hamda Jama Yousuf, Abdiasis Aden Omer, Bashir Mohamed Mohamoud, Ahmed Abdirahman Farah, Ali Ahmed Hussein, Fatima Nouh Ismail, Mohamed Hussein Egeh

**Affiliations:** 1 Research Department, Ogaansho Research and Consultancy Centre, Burao, Somaliland; 2 Department of Education, Faculty of Education, University of Burao, Burao, Somaliland; 3 School of Postgraduate Studies and Research, Abaarso Tech University, Burao, Somaliland

**Keywords:** antenatal care, counseling, Demographic and Health Survey, maternal health, neonatal danger signs, Somalia

## Abstract

**Background:**

Neonatal mortality remains a major public health challenge in low- and middle-income countries, including Somalia, where access to maternal health services is limited. Although counseling on neonatal danger signs is essential for early recognition and timely care-seeking, evidence on its coverage and determinants in Somalia remains scarce, particularly using nationally representative data.

**Methods:**

A cross-sectional study was conducted using data from the 2020 Somalia Demographic and Health Survey (SDHS). A total of 6,413 women aged 15–49 years with a recent birth were included. A multilevel mixed-effects logistic regression model was employed to identify individual- and community-level determinants of receiving counseling. Adjusted Odds Ratios (AORs) with 95% Confidence Intervals (CIs) were reported.

**Results:**

Only 4.35% of women reported receiving counseling on neonatal danger signs. Women in the highest wealth quintile were significantly more likely to receive counseling (AOR = 2.77; 95% CI: 1.60–4.80). Adequate antenatal care utilization was associated with increased odds (AOR = 2.10; 95% CI: 1.40–3.13), while facility-based delivery was the strongest predictor (AOR = 4.49; 95% CI: 3.28–6.14).

**Conclusion:**

Counseling on neonatal danger signs in Somalia remains critically low and is characterized by substantial socioeconomic and healthcare access inequalities. Strengthening antenatal care services, promoting facility-based delivery, and addressing equity gaps in access to maternal health services are essential to improve maternal knowledge and neonatal outcomes.

## Background

Neonatal mortality, defined by the World Health Organization as death within the first 28 days of life, is a fundamental indicator of newborn health and survival. It reflects the overall socioeconomic status of a country as well as the accessibility and quality of maternal and child health services. High neonatal mortality rates are often associated with inadequate maternal and newborn care, particularly limited access to timely and effective healthcare interventions during pregnancy, childbirth, and the early postnatal period. However, most causes of neonatal death are preventable through appropriate maternal and newborn healthcare services (1).

Neonates represent the most vulnerable population group, requiring specialized care due to their physiological immaturity and increased susceptibility to illness. Neonatal danger signs are critical clinical indicators of severe illness that can be recognized by mothers and other caregivers. Early recognition of these signs is essential for prompt care-seeking, which can significantly reduce neonatal morbidity and mortality. Globally, an estimated 2.5 million neonates died in 2022, with the majority of deaths occurring in low- and middle-income countries. Sub-Saharan Africa and South Asia account for the highest burden of neonatal mortality. A child born in these regions is more than ten times more likely to die in the first month of life compared to a child born in high-income countries. Despite global efforts to reduce neonatal mortality, many deaths still occur at home, where caregivers often lack adequate knowledge of neonatal danger signs and access to timely healthcare services (2).

Counseling on neonatal danger signs is a key component of maternal and newborn healthcare, particularly during the immediate postnatal period. Effective counseling enables mothers to recognize early signs of illness and seek timely medical care. However, the effectiveness of counseling depends not only on its availability but also on the quality, clarity, and retention of information provided by healthcare providers. In many low-resource settings, opportunities for such counseling remain limited, especially among women with restricted access to healthcare services (3). Despite the critical importance of counseling on neonatal danger signs, evidence on its coverage and determinants in Somalia remains limited, particularly using nationally representative data. Moreover, most existing studies focus on antenatal counseling, with less attention given to counseling provided during the immediate postnatal period, which is a crucial window for improving newborn survival. Therefore, this study aimed to investigate the individual- and community-level determinants of receiving counseling on neonatal danger signs among women in Somalia, using data from the 2020 Somalia Demographic and Health Survey. Addressing this evidence gap is essential for improving maternal and neonatal health outcomes and aligns with the United Nations Sustainable Development Goals (SDGs), particularly SDG 3, which seeks to ensure healthy lives and promote well-being for all at all ages. Specifically, this study contributes to Target 3.2, which aims to reduce neonatal mortality, and Target 3.8, which focuses on achieving universal health coverage through improved access to quality maternal and newborn healthcare services.

## Methods and material

This study used data from the 2020 Somalia Demographic and Health Survey (SDHS), a nationally representative cross-sectional survey designed to collect information on population health, including maternal and child health indicators. The SDHS employed a stratified, two-stage cluster sampling design to ensure representativeness across urban, rural, and nomadic populations. In the first stage, Enumeration Areas (EAs), serving as Primary Sampling Units (PSUs), were selected using probability proportional to size from a nationally defined sampling frame. In the second stage, households were systematically selected within each EA. All women aged 15–49 years who were usual residents or visitors in the selected households were eligible for the survey (4). For this study, the analysis was restricted to women who had a live birth within the five years preceding the survey. Observations with missing data on the outcome variable and key covariates were excluded. This resulted in a final weighted analytical sample of 6,413 women.

### Study variables

#### Outcome variable

The outcome variable was receipt of counseling on neonatal danger signs, defined as whether a woman reported receiving counseling from a health provider on neonatal danger signs within the first two days after delivery. The variable was coded as a binary outcome (1 = Yes, 0 = No).

### Independent variables

Independent variables were categorized into individual-level and community-level factors. The individual-level variables included maternal age (15–49 years), educational level (no education, primary, secondary, and higher), employment status (yes/no), wealth index (poorest to richest quintile), and household size (<4 and ≥4 members). Reproductive and behavioral characteristics such as parity (primipara, multipara, and grand multipara), age at first birth (<20, 20–24, 25–30, and ≥30 years), and pregnancy intention (wanted then, wanted later, and no more) were also included. In addition, variables related to access and decision-making were considered, including media exposure (yes/no), healthcare decision-making autonomy (self, joint, and others), perceived financial barriers to healthcare (money for treatment: big problem/not a problem), and perceived distance to a health facility (big problem/not a problem). Utilization of maternal health services was assessed using antenatal care (ANC) utilization, which was categorized into three groups based on the number of visits: no ANC (0 visits), partial ANC (1–3 visits), and adequate ANC (≥4 visits), in line with previous DHS-based studies and WHO recommendations applicable at the time of the survey, and place of delivery (home vs. health facility). At the community level, variables included place of residence (urban, rural, and nomadic) and region.

### Data processing and analysis

Data were extracted from the 2020 Somalia Demographic and Health Survey (SDHS) and analyzed using Stata version 17. Before analysis, the dataset was cleaned to ensure data quality by checking for missing values, inconsistencies, and outliers. Observations with incomplete information on key variables were excluded from the final analysis. To account for the complex survey design, sampling weights, clustering, and stratification were applied. To account for the complex survey design of the SDHS, sampling weights, clustering, and stratification were applied during the analysis. The individual sampling weight variable (V005) was normalized by dividing by 1,000,000 and applied to adjust for unequal probabilities of selection and non-response. The multilevel mixed-effects logistic regression model additionally accounted for clustering at the primary sampling unit (PSU) level through random intercept specification. Descriptive statistics were used to summarize the characteristics of the study population, and results were presented using frequencies and percentages. Bivariate analysis was conducted to assess the association between independent variables and the outcome. Variables with a p-value <0.25 in the bivariate analysis were considered for inclusion in the multivariable model. In addition, variables identified as theoretically important and consistently associated with maternal healthcare utilization in previous literature were retained regardless of their bivariate statistical significance. To account for the hierarchical structure of the data, where individuals are nested within clusters, a multilevel mixed-effects logistic regression model was employed. Four models were fitted: the null model (without explanatory variables), Model I (individual-level variables), Model II (community-level variables), and Model III (both individual- and community-level variables). Model comparison was conducted using Akaike Information Criterion (AIC), Bayesian Information Criterion (BIC), and log-likelihood values. The final model was selected based on a combination of statistical fit (lower AIC and higher log-likelihood) and theoretical relevance of variables. Adjusted Odds Ratios (AOR) with 95% Confidence Intervals (CI) were reported to identify factors associated with counseling on neonatal danger signs. Statistical significance was declared at a p-value less than 0.05. In addition, the intra-class correlation coefficient (ICC) for the multilevel logistic regression models was calculated using the latent variable approach, assuming a level-1 residual variance of π^2^/3 (3.29). The ICC was estimated using the formula: ICC = Va/(Va + 3.29), where Va represents the cluster-level variance.

## Result

### Prevalence counseling on neonatal danger signs

The prevalence of counseling on neonatal danger signs among women in Somalia was found to be very low. Only 4.35% of women reported receiving counseling within the first two days after delivery, while the vast majority (95.65%) did not receive such counseling. This finding highlights a substantial gap in early postnatal health education and indicates missed opportunities for improving maternal awareness of neonatal danger signs.

### Sociodemographic characteristics of the study participants

The sociodemographic and obstetric characteristics of women of reproductive age in Somalia, based on the 2020 SDHS, indicate a predominantly young and socioeconomically disadvantaged population. The largest proportion of women was aged 25–29 years (28.34%), followed by those aged 20–24 years (21.26%) and 30–34 years (19.55%), while only a small fraction was aged 45–49 years (1.84%). Regionally, the highest representation was from Banadir (22.70%), followed by Woqooyi Galbeed (10.12%), whereas Awdal (2.13%) and Bakool (2.70%) had the lowest proportions.

In terms of residence, the majority of women lived in urban areas (60.32%), with smaller proportions residing in rural (25.57%) and nomadic settings (14.11%). Educational attainment was notably low, with the vast majority of women having no formal education (84.07%), while only a small proportion had secondary (2.96%) or higher education (0.83%). Most households were large, with 79.79% of women living in households with four or more members. The wealth distribution showed that 26.02% of women belonged to the poorest quintile, whereas only 15.94% were in the highest wealth category. Regarding reproductive characteristics, a substantial proportion of women were multiparous (45.30%) or grand multiparous (42.55%), and more than half (54.32%) had their first birth before the age of 20. Financial and geographic barriers to healthcare were highly prevalent, with 68.88% reporting that obtaining money for treatment was a major problem and 64.98% indicating that distance to a health facility was a significant challenge. Employment among women was extremely low (0.80%), and decision-making regarding healthcare was largely dominated by others (46.57%), with only 21.38% of women making decisions independently.

Furthermore, most pregnancies were intended at the time (64.39%), although a notable proportion were mistimed (26.74%) or unwanted (8.87%). Utilization of antenatal care (ANC) services was low, with 67.81% of women not utilizing ANC at all, and only 8.29% receiving adequate care. Similarly, the majority of deliveries occurred at home (76.84%), while only 23.16% took place in health facilities. In addition, media exposure was limited, with 62.06% of women reporting no exposure to media. Overall, these findings highlight significant socioeconomic, geographic, and healthcare access challenges among women in Somalia. The sociodemographic and obstetric characteristics of the study participants are presented in Table 1.

**Table 1:** Weighted sociodemographic and obstetric characteristics of women of reproductive Age in Somalia, sDHS 2020 (*n* = 6,413).

Variables	Category	Weighted n	Percent (%)
Age group (years)	15–19	413	6.81
	20–24	1,291	21.26
	25–29	1,721	28.34
	30–34	1,187	19.55
	35–39	956	15.73
	40–44	393	6.48
	45–49	112	1.84
Region	Awdal	130	2.13
	Woqooyi Galbeed	615	10.12
	Togdheer	322	5.30
	Sool	206	3.39
	Sanaag	251	4.13
	Bari	407	6.70
	Nugaal	192	3.17
	Mudug	445	7.33
	Galgaduud	375	6.17
	Hiraan	229	3.77
	Middle Shabelle	408	6.72
	Banadir	1,379	22.70
	Bay	334	5.49
	Bakool	164	2.70
	Gedo	259	4.27
	Lower Juba	359	5.91
Place of residence	Rural	1,553	25.57
	Urban	3,664	60.32
	Nomadic	857	14.11
Educational level	No education	5,107	84.07
	Primary	737	12.13
	Secondary	180	2.96
	Higher	51	0.83
Household size	<4 members	1,228	20.21
	≥4 members	4,846	79.79
Wealth index	Lowest	1,580	26.02
	Second	1,225	20.17
	Middle	1,093	18.00
	Fourth	1,207	19.86
	Highest	968	15.94
Parity	Primipara	738	12.15
	Multipara	2,752	45.30
	Grand multipara	2,584	42.55
Age at first birth	<20	3,299	54.32
	20–24	2,094	34.48
	25–30	588	9.68
	≥30	93	1.53
Money for treatment	Big problem	4,184	68.88
	Not a problem	1,890	31.12
Distance to the health facility	Big problem	3,947	64.98
	Not a problem	2,127	35.02
Employment	Yes	49	0.80
	No	6,025	99.20
Healthcare decision-making	Self	1,299	21.38
	Together	1,946	32.05
	Others	2,829	46.57
Pregnancy intention	Then	3,911	64.39
	Later	1,624	26.74
	No more	539	8.87
ANC utilization	Not utilized	4,119	67.81
	Partial	1,452	23.90
	Adequate	503	8.29
Place of delivery	Home	4,668	76.84
	Facility	1,406	23.16
Media exposure	No	3,770	62.06
	Yes	2,304	37.94

Minor differences in weighted frequencies across variables are due to variable-specific missing values and rounding adjustments after applying survey weights.

### Bivariate analysis of factors associated with counseling on neonatal danger signs among women in Somalia, 2020

The bivariate logistic regression analysis identified several factors significantly associated with counseling on neonatal danger signs among women in Somalia. There was no statistically significant association between maternal age and counseling (*χ*^2^ = 0.69, *p* = 0.609), indicating that counseling uptake did not vary meaningfully across age groups. Similarly, place of residence (*χ*^2^ = 0.12, *p* = 0.858), household size (*χ*^2^ = 0.04, *p* = 0.837), parity (*χ*^2^ = 1.27, *p* = 0.281), age at first birth (*χ*^2^ = 0.94, *p* = 0.405), employment status (*χ*^2^ = 1.50, *p* = 0.222), decision-making autonomy (*χ*^2^ = 1.01, *p* = 0.361), pregnancy intention (*χ*^2^ = 2.36, *p* = 0.095), and media exposure (*χ*^2^ = 0.88, *p* = 0.349) were not significantly associated with receiving counseling.

In contrast, several variables demonstrated statistically significant associations with counseling. Regional variation was significant (*χ*^2^ = 2.90, *p* = 0.002), indicating disparities in counseling coverage across different regions of Somalia. Educational level showed a strong association (*χ*^2^ = 16.25, *p* < 0.001), with women who had higher levels of education being more likely to receive counseling compared to those with no formal education. Similarly, wealth index was significantly associated with counseling (*χ*^2^ = 25.03, *p* < 0.001), as women from higher wealth quintiles had a greater proportion of counseling uptake than those from poorer households. Access-related barriers also played a critical role. Women who reported that obtaining money for treatment was not a major problem were more likely to receive counseling compared to those who faced financial constraints (*χ*^2^ = 24.21, *p* < 0.001). Likewise, distance to a health facility was significantly associated with counseling (*χ*^2^ = 40.86, *p* < 0.001), with women who did not perceive distance as a major barrier having higher counseling coverage. Utilization of maternal health services emerged as one of the strongest determinants. Antenatal care (ANC) visits were significantly associated with counseling (*χ*^2^ = 30.17, *p* < 0.001), as women who had partial or adequate ANC visits were more likely to receive counseling compared to those who did not utilize ANC services. Furthermore, the place of delivery showed the strongest association (*χ*^2^ = 111.73, *p* < 0.001), with women who delivered in health facilities being substantially more likely to receive counseling than those who delivered at home.

Overall, these findings suggest that socioeconomic status, access to healthcare, and utilization of maternal health services are key factors influencing counseling on neonatal danger signs, while most demographic characteristics were not significantly associated at the bivariate level. The bivariate logistic regression results are presented in Table 2.

**Table 2:** Bivariate logistic regression analysis of factors associated with counseling on neonatal danger signs among women in Somalia, 2020.

Variable	Category	Counseling on newborn danger signs		*χ*^2^ (p-value)
		Yes n (%)	No n (%)	
Age group	15–19	24 (5.89)	389 (94.11)	0.69 (0.609)
	20–24	83 (20.55)	1208 (79.45)	
	25–29	132 (32.58)	1589 (67.42)	
	30–34	88 (21.63)	1,099 (78.37)	
	35–39	58 (14.32)	898 (85.68)	
	40–44	15 (3.69)	378 (96.31)	
	45–49	5 (1.34)	107 (98.66)	
Region	Awdal	14 (3.55)	116 (96.45)	2.90 (0.002)
	Woqooyi Galbeed	27 (6.58)	588 (93.42)	
	Togdheer	28 (6.90)	294 (93.10)	
	Sool	16 (3.94)	190 (96.06)	
	Sanaag	18 (4.49)	233 (95.51)	
	Bari	17 (4.22)	390 (95.78)	
	Nugaal	11 (2.74)	181 (97.26)	
	Mudug	12 (2.88)	433 (97.12)	
	Galgaduud	14 (3.61)	361 (96.39)	
	Hiraan	27 (6.89)	202 (93.11)	
	Middle Shabelle	45 (11.65)	363 (88.35)	
	Banadir	104 (26.88)	1,275 (73.12)	
	Bay	4 (1.11)	330 (98.89)	
	Bakool	26 (6.98)	138 (93.02)	
	Gedo	6 (1.61)	253 (98.39)	
	Lower Juba	23 (5.95)	336 (94.05)	
Residence	Rural	104 (26.51)	1,449 (73.49)	0.12 (0.858)
	Urban	239 (60.87)	3,425 (39.13)	
	Nomadic	50 (12.62)	807 (87.38)	
Education	No education	276 (70.58)	4,831 (29.42)	16.25 (<0.001)
	Primary	72 (18.39)	665 (81.61)	
	Secondary	26 (6.76)	154 (93.24)	
	Higher	16 (4.27)	35 (95.73)	
Household size	<4	82 (20.81)	1,146 (79.19)	0.04 (0.837)
	≥4	312 (79.19)	4,534 (20.81)	
Wealth index	Poorest	31 (7.98)	1,549 (92.02)	25.03 (<0.001)
	Second	39 (10.13)	1,186 (89.87)	
	Middle	56 (14.58)	1,037 (85.42)	
	Fourth	102 (26.53)	1,105 (73.47)	
	Highest	166 (40.78)	802 (59.22)	
Parity	Primipara	64 (16.25)	674 (83.75)	1.27 (0.281)
	Multipara	172 (43.70)	2,580 (56.30)	
	Grand multipara	158 (40.04)	2,426 (59.96)	
Age at first birth	<20	213 (54.17)	3,086 (45.83)	0.94 (0.405)
	20–24	124 (31.68)	1,970 (68.32)	
	25–30	52 (13.20)	536 (86.80)	
	≥30	4 (0.95)	89 (99.05)	
Money for treatment	Big problem	209 (53.20)	3,975 (46.80)	24.21 (<0.001)
	Not a problem	184 (46.80)	1,706 (53.20)	
Distance to the health facility	Big problem	171 (43.60)	3,776 (56.40)	40.86 (<0.001)
	Not a problem	222 (56.40)	1,905 (43.60)	
Employment	Yes	7 (1.68)	42 (98.32)	1.50 (0.222)
	No	387 (98.32)	5632 (1.68)	
Decision-making	Self	85 (21.69)	1,214 (78.31)	1.01 (0.361)
	Together	143 (36.47)	1,803 (63.53)	
	Others	164 (41.84)	2,665 (58.16)	
Pregnancy intention	Then	279 (70.90)	3,632 (29.10)	2.36 (0.095)
	Later	88 (22.39)	1,536 (77.61)	
	No more	26 (6.71)	513 (93.29)	
ANC utilization	Not utilized	158 (40.15)	3,961 (59.85)	30.17 (<0.001)
	Partial	155 (39.32)	1,297 (60.68)	
	Adequate	81 (20.53)	422 (79.47)	
Place of delivery	Home	145 (36.80)	4,523 (63.20)	111.73 (<0.001)
	Facility	249 (63.20)	1,157 (36.80)	
Media exposure	No	229 (58.16)	3,541 (41.84)	0.88 (0.349)
	Yes	164 (41.84)	2,140 (58.16)	

*p*-values are based on the Pearson chi-square test adjusted for survey design.

### Multilevel mixed-effects logistic regression models for counseling in Somalia, 2020

The multilevel mixed-effects logistic regression results are shown in Table 3 and Table 4. The model comparison of multilevel mixed-effects logistic regression models demonstrated that the full model (Model III), which included both individual- and community-level variables, provided the best fit to the data. This is supported by its highest log-likelihood value (−960.705), indicating improved explanatory power compared to the null model (−1137.557), Model I (−985.030), and Model II (−1115.697). Furthermore, Model III had the lowest Akaike Information Criterion (AIC = 2017.409), suggesting the best balance between model fit and complexity among all models. Although Model I also showed a relatively low AIC (2034.059), it remained higher than that of Model III, indicating comparatively poorer model performance. While the Bayesian Information Criterion (BIC) was lowest for the null model (2292.647), model selection was primarily based on AIC and log-likelihood, which are more appropriate for comparing nested models in this context. Additionally, Model III demonstrated superior explanatory capacity by incorporating both individual- and community-level determinants. The likelihood ratio (LR) test further indicated that Model III provided a statistically significant improvement over simpler models (*p* < 0.001). The intra-class correlation coefficient (ICC) in the null model (10.63%) indicates that a considerable proportion of the variation in counseling on neonatal danger signs was attributable to differences between clusters, supporting the use of a multilevel modeling approach. Model III was selected as the best-fitting model because it had the lowest AIC value and the highest log-likelihood compared to the other models.

**Table 3:** Model comparison of multilevel mixed-effects logistic regression models for counseling on neonatal danger signs among women in Somalia, 2020.

Parameters	Null Model	Model I	Model II	Model III
Log-likelihood	−1,137.557	−985.030	−1,115.697	−960.705
AIC	2,279.115	2,034.059	2,269.393	2,017.409
BIC	2,292.647	2,250.574	2,397.949	2,342.181
LR test	19.83	0.67	11.67	0.00
p-value	<0.001	0.206	<0.001	<0.001
ICC(%)	10.63	2.32	7.05	7.00

ICC, Intraclass Correlation Coefficient; AIC, Akaike Information Criterion; BIC, Bayesian Information Criterion.

**Table 4:** Multilevel mixed-effects logistic regression analysis of factors associated with counseling on neonatal danger signs among women in Somalia: evidence from the 2020 survey (model III).

Variable	Category	Model III AOR (95% CI)
Age (years)	15–19	Ref
	20–24	1.17 (0.65–2.14)
	25–29	1.34 (0.69–2.57)
	30–34	1.45 (0.69–3.03)
	35–39	1.39 (0.62–3.09)
	40–44	1.30 (0.48–3.47)
	45–49	1.39 (0.37–5.27)
Education level	No education	Ref
	Primary	0.92 (0.65–1.30)
	Secondary	0.94 (0.56–1.57)
	Higher	1.63 (0.81–3.30)
Working status	Yes	Ref
	No	0.73 (0.21–2.50)
Media exposure	No	Ref
	Yes	0.92 (0.68–1.24)
Wealth index	Poorest	Ref
	Second	1.09 (0.60–1.98)
	Middle	1.48 (0.85–2.59)
	Fourth	1.78 (1.02–3.08)*
	Highest	2.77 (1.60–4.80)***
Household size	<4	Ref
	≥4	0.97 (0.72–1.32)
Parity	Primipara	Ref
	Multipara	0.75 (0.50–1.12)
	Grand multipara	0.72 (0.42–1.23)
Age at first birth	<20	Ref
	20–24	0.80 (0.59–1.10)
	25–30	1.22 (0.74–2.00)
	≥30	0.98 (0.33–2.88)
Pregnancy intention	Then	Ref
	Later	0.85 (0.61–1.16)
	No more	1.07 (0.65–1.78)
Money for treatment	Big problem	Ref
	Not a problem	1.12 (0.79–1.58)
Distance to the health facility	Big problem	Ref
	Not a problem	1.45 (1.02–2.07)*
Decision-making	Alone	Ref
	Together	0.99 (0.70–1.42)
	Others	1.01 (0.72–1.44)
ANC utilization	Not utilized	Ref
	Partially utilized	1.61 (1.17–2.21)**
	Adequately utilized	2.10 (1.40–3.13)***
Place of delivery	Home	Ref
	Health facility	4.49 (3.28–6.14)***
Region	Awdal	Ref
	Woqooyi Galbeed	1.22 (0.60–2.49)
	Togdheer	0.98 (0.49–1.95)
	Sool	1.00 (0.50–1.98)
	Sanaag	0.98 (0.48–1.98)
	Bari	1.60 (0.69–3.71)
	Nugaal	2.31 (1.07–5.00)*
	Mudug	0.99 (0.37–2.69)
	Galgaduud	1.30 (0.52–3.22)
	Hiraan	2.27 (1.05–4.90)
	Middle Shabelle	1.36 (0.65–2.86)
	Banadir	1.39 (0.70–2.75)
	Bay	0.23 (0.03–1.80)
	Bakool	3.88 (2.00–7.55)***
	Gedo	1.44 (0.53–3.96)
	Lower Juba	1.96 (0.90–4.27)
Residence	Rural	Ref
	Urban	1.47 (1.01–2.12)*
	Nomadic	1.39 (0.97–1.99)

AOR, Adjusted Odds Ratio; CI,  Confidence Interval; Ref, Reference category.

* *p* < 0.05.

** *p* < 0.01.

*** *p* < 0.001.

The multilevel mixed-effects logistic regression analysis identified several significant determinants of counseling on neonatal danger signs among women in Somalia. After adjusting for both individual- and community-level factors (Model III), socioeconomic status, healthcare access, service utilization, and geographic factors remained key predictors.

Women from higher wealth quintiles had significantly greater odds of receiving counseling compared to those from the poorest households. Specifically, women in the fourth wealth quintile were 1.78 times more likely (AOR = 1.78; 95% CI: 1.02–3.08), and those in the highest wealth quintile were 2.77 times more likely (AOR = 2.77; 95% CI: 1.60–4.80) to receive counseling. Similarly, accessibility to healthcare services played a significant role; women who reported that distance to a health facility was not a major problem had higher odds of receiving counseling (AOR = 1.45; 95% CI: 1.02–2.07). Utilization of maternal health services was strongly associated with counseling. Women who partially utilized antenatal care (ANC) services were 1.61 times more likely (AOR = 1.61; 95% CI: 1.17–2.21), while those who adequately utilized ANC were more than twice as likely (AOR = 2.10; 95% CI: 1.40–3.13) to receive counseling compared to women who did not utilize ANC. Furthermore, the place of delivery was the strongest predictor identified in the model. Women who delivered in health facilities had significantly higher odds, approximately 4.5 times more likely (AOR = 4.49; 95% CI: 3.28–6.14) to receive counseling compared to those who delivered at home.

Geographic disparities were also evident. Women residing in urban areas were significantly more likely to receive counseling than those in rural areas (AOR = 1.47; 95% CI: 1.01–2.12). Additionally, significant regional variations were observed. Women from Nugaal (AOR = 2.31; 95% CI: 1.07–5.00), Hiraan (AOR = 2.27; 95% CI: 1.05–4.90), and Bakool (AOR = 3.88; 95% CI: 2.00–7.55) had significantly higher odds of receiving counseling compared to those from Awdal. In contrast, several variables were not significantly associated with counseling in the adjusted model. These included maternal age, educational level, employment status, media exposure, household size, parity, age at first birth, pregnancy intention, financial barriers, and healthcare decision-making autonomy, as their confidence intervals included unity.

Overall, the findings indicate that counseling on neonatal danger signs in Somalia is primarily influenced by socioeconomic status, access to healthcare, utilization of maternal health services, and geographic location, rather than individual demographic characteristics.

## Discussion

Using nationally representative data from the 2020 survey, this study investigated the prevalence and factors influencing counseling on neonatal danger signs among women in Somalia of reproductive age. The results showed that counseling coverage is still low, with only 4.35% of women reporting that they received counseling on neonatal danger signs within the first two days after delivery, while the vast majority (95.65%) did not receive such counseling. This indicates substantial gaps in maternal and newborn health education during the immediate postnatal period.

The prevalence observed in this study is considerably lower than that reported in other settings, including China (42%) (5)., Kenya (51.8%) (6), Southern Ethiopia (35.5%) (7), West-central Ethiopia (65.5%) (8), and Northwest Ethiopia (64.5%) (9). All of these discrepancies may result from differences in the sociodemographic traits of research participants as well as aspects of the health system (such as the lack of healthcare providers and distance to Health facilities). Additionally, the discrepancy may also result from variations in the study setting, time, and participants (the current finding was focused on the uptake of health information in the immediate postnatal period). In contrast to UNICEF's recommendation that all pregnant women receive counseling regarding these warning signs and immediate postnatal period, the uptake was insufficient.

Socioeconomic status was identified as an important determinant of counseling. Women from higher wealth quintiles were significantly more likely to receive counseling compared to those from poorer households. This finding is consistent with studies conducted in Papua New Guinea (10), India (11), and Bangladesh (12). which demonstrated that wealthier women are more likely to benefit from maternal health education and have better access to healthcare services. Financially capable women can get past obstacles like service fees and transportation costs, which increases their access to qualified healthcare professionals. Utilization of maternal health services was also strongly associated with receiving counseling. Women who attended antenatal care (ANC) visits, whether partially or adequately, were significantly more likely to receive counseling compared to those who did not utilize ANC services. This is in line with data from multiple studies of Middle-income countries (13), Kenya (14), Uganda (15) and Madagascar (16). which highlight the important role of ANC as a platform for delivering essential maternal and newborn health information. Increased utilization of ANC services may improve maternal awareness and promote timely care-seeking behavior.

Place of delivery emerged as the strongest predictor of counseling. Women who delivered in health facilities were substantially more likely to receive counseling compared to those who delivered at home. This finding is supported by studies conducted in Ethiopia (17–19) and Ghana (20), where facility-based delivery is closely linked to improved access to postnatal care and maternal education. In the present study, the high proportion of home deliveries (76.84%) represents a major barrier to the provision of counseling services, as women delivering at home have limited interaction with skilled healthcare providers. Geographic disparities were also evident. Women residing in urban areas were more likely to receive counseling than those in rural areas, which may reflect differences in access to healthcare infrastructure, availability of skilled health workers, and exposure to health information. Additionally, regional variations suggest inequalities in the distribution of healthcare services and resources across the country (21, 22). Furthermore, regional differences found in this study point to disparities in the provision of healthcare services between various regions, most likely as a result of differences in the capacity of the health system and the distribution of resources as reported previously (23).

Overall, the findings highlight critical gaps in maternal and neonatal health education in Somalia. Strengthening antenatal care services, promoting facility-based delivery, and improving access to healthcare services, particularly in rural and underserved areas, are essential strategies for increasing counseling coverage. Targeted interventions focusing on disadvantaged populations are crucial for improving maternal knowledge and reducing neonatal morbidity and mortality.

## Conclusion

This study revealed that counseling on neonatal danger signs among women in Somalia remains critically low, with only a very small proportion of mothers receiving counseling within the early postnatal period. The findings demonstrated that counseling is strongly influenced by socioeconomic status, access to healthcare services, and utilization of maternal health services, particularly antenatal care attendance and facility-based delivery. Women from wealthier households, those with better geographic access to health facilities, and those who utilized ANC and delivered in health institutions were significantly more likely to receive counseling.

The study further highlights substantial inequalities in counseling coverage across urban–rural settings and regions, reflecting broader disparities in healthcare access and service delivery. These findings underscore the urgent need for strengthening maternal and neonatal health services in Somalia. Interventions aimed at improving ANC utilization, promoting facility-based delivery, and reducing geographic and financial barriers to healthcare are essential to enhance counseling coverage.

Policymakers and health program planners should prioritize community-based interventions, outreach programs, and health system strengthening strategies to ensure that all women especially those in rural and underserved areas have access to essential maternal health information. Improving counseling on neonatal danger signs is crucial for early recognition of neonatal complications and can play a significant role in reducing neonatal morbidity and mortality in Somalia.

## Data Availability

The original contributions presented in the study are included in the article/Supplementary Material, further inquiries can be directed to the corresponding author.
